# Nutritional modulation of disease severity in acute pancreatitis: metabolic pathways, inflammatory signaling, and diet-responsive clinical outcomes

**DOI:** 10.3389/fnut.2026.1789279

**Published:** 2026-07-01

**Authors:** Zhongli Wang, Yangjun Liu, Chi Li

**Affiliations:** Second Ward of Hepatobiliary Surgery, General Surgery Department, The First Affiliated Hospital of Jinzhou Medical University, Jinzhou, China

**Keywords:** acute pancreatitis, albumin, enteral nutrition, nutritional status, protein intake, sarcopenia, vitamin D deficiency

## Abstract

**Background:**

The severity of acute pancreatitis (AP) is influenced by metabolic stress, systemic inflammation, gut barrier dysfunction, and nutritional status. While supportive care remains central to management, growing evidence indicates that nutritional modulation impacts disease severity, organ failure, and mortality.

**Methods:**

A retrospective study of 1,600 AP patients, diagnosed using the Revised Atlanta Criteria, was conducted to evaluate demographics, nutritional status, metabolic and inflammatory biomarkers, dietary patterns, gut barrier markers, and clinical outcomes. Early (≤48 h) versus delayed enteral nutrition (EN) was analyzed. Multivariable logistic regression adjusted for age, sex, BMI, etiology, and comorbidities was used to identify independent nutritional predictors of severe disease, persistent organ failure, and in-hospital mortality.

**Results:**

Age, BMI, and APACHE II score were major contributors to severity (APACHE II 16.8 ± 5.1 in severe vs. 6.8 ± 3.1 in mild AP; *p* < 0.001). Severe AP was characterized by marked nutritional depletion, including hypoalbuminemia (2.92 ± 0.69 g/dL), sarcopenia (53.9%), vitamin D deficiency (72.2%), and hypertriglyceridemia (54.4%) (all *p* < 0.001). Early EN significantly reduced systemic inflammation (CRP: 88 ± 46 vs. 142 ± 62 mg/L; IL-6: 29.6 ± 13.2 vs. 48.9 ± 19.6 pg./mL) and increased protein intake (1.12 ± 0.29 vs. 0.78 ± 0.32 g/kg/day). It also preserved gut barrier integrity and reduced pancreatic necrosis (9.1% vs. 24.7%, both *p* < 0.001). Metabolic assessment revealed progressive insulin resistance (HOMA-IR 5.3 ± 2.1), elevated lactate (3.2 ± 0.9 mmol/L), and mitochondrial dysfunction in severe AP. High-fat and low-fiber diets doubled the risk of severity (OR 2.61–2.78), whereas omega-3 intake, Mediterranean diet adherence, and vitamin D sufficiency were protective (OR 0.43–0.53). Early EN reduced the odds of severe disease by 56% (OR 0.44, 95% CI 0.34–0.58).

**Conclusion:**

Nutritional modulation substantially affects metabolic, inflammatory, and clinical trajectories in AP, supporting early targeted nutrition as a core therapeutic strategy.

## 1 Introduction

Acute pancreatitis (AP) is an acute inflammatory disease of the pancreas with a wide clinical presentation of mild, self-limiting disease to severe acute pancreatitis (SAP) with systemic inflammatory response syndrome (SIRS) and dysfunction of multiple organs and a high death rate (1). Although the supportive care is progressing, the world is getting exposed to the increasing incidence of AP, mostly caused by metabolic risk factors, including obesity, hypertriglyceridemia, alcohol use, and diet (2). There is growing evidence that nutritional status and dietary interventions are not merely passive aspects of care but active regulators of disease severity andhave changed this paradigm and proved that early enteral nutrition (EN) maintains gut integrity, suppresses systemic inflammation, and yields positive clinical outcomes (3, 4). These data highlight the growing importance of nutrition as a therapeutic instrument that affects metabolic, inflammatory, and immune pathways in the event of acute pancreatic injury. Host nutritional status, metabolic disruptions, the gut microbiota, and immune responses interact in complex ways to determine the severity of AP. All three have been implicates, individually in the severity of disease, increased rates of complications, and extended hospitalization (5). Dysfunction in adipose tissue, especially in obese patients, contributes to excess free fatty acid and adipokine secretion, worsening of pancreatic necrosis, and systemic inflammation.

Recent meta-design evidence has shown that adipokines (leptin, adiponectin), this can be used to predict the severity of AP, supporting a correlation between the nutritional-metabolic condition and the degree of inflammation (6). Another clinically relevant concept, feeding intolerance, has been observed to be a predictor and contributor to poorer outcomes, often manifesting as impaired gut motility, dysbiosis, and hyperinflammatory signaling (7). In addition to the balance of macronutrients, changes in the gut microbiota driven by diet have now become key determinants of pancreatic inflammation. Theis et al. (8) also highlight that diet-microbiome-host immune interactions have a profound influence on the development of systemic inflammatory diseases, a conceptual framework that is highly applicable to the pathophysiology of AP. Similarly, recent gut-pancreas axis studies show that microbial metabolites and intestinal barrier dysfunction have the potential to mediate pancreatic inflammation and disrupt metabolic homeostasis (9).

At the mechanistic level, AP is driven by metabolic dysregulation, mitochondrial dysfunction, and dysregulated inflammatory signaling. According to recent metabolomic investigations, amino acid metabolism changes are pronounced in AP, especially in the tryptophan-kynurenine pathway. Wang et al. (10) have shown that immune dysregulation and a higher tendency to infection are tightly associated with altered tryptophan metabolism in the course of AP, indicating a diet-responsive metabolic pathway that contributes to the severity of the disease. NF-κB, AMPK, and cytokine networks, among other inflammatory signaling cascades, are key mediators of the enhancement of pancreatic and systemic inflammation. Wen et al. (11) presented strong evidence supporting the idea that gut microbiota modulation with *Clostridium butyricum* can regulate the AMPK/NF-κB signaling through metabolomic alterations, leading to decreased inflammation and pancreatic injury. These results are consistent with the general ideas of Theis et al. (8), according to which microbial metabolites can be considered one of the main intermediate factors between diet and host immunity. Mitochondrial dysfunction and impaired mitophagy have also been shown to be critical at the cellular level in causing injury to acinar cells. Zhu et al. (12) emphasized that impaired mitophagy increases oxidative stress and inflammatory signals in AP and additionally linked disease development to energy metabolism. Nutritional treatments involving mitochondrial activity and oxidative equilibrium can thus be unexploited treatment modalities.

The clinical recommendations strongly support early enteral nutrition, preferably via gastric or jejunal tubing, because of its role in reducing infectious complications and mortality (4). In addition to timing and route, nutritional composition, such as the quality of lipids, fiber, probiotics, and immune-nutrients, has been shown to alter inflammatory responses and metabolism (2, 3). However, even with growing evidence, there has been considerable heterogeneity in clinical response to nutritional interventions, in part because of variable baseline nutritional status, metabolic phenotype, and gut microbiota composition. Such heterogeneity brings to light the need for a more personalized, mechanism-based approach to nutrition in AP management.

Even though there is significant progress that has been achieved in terms of the role of nutrition in AP, there are still a few gaps that are essential. Few studies have incorporated metabolic, microbial, and inflammatory processes, with most relying on clinical outcomes, including mortality or hospital stay. Also, although research has been performed to investigate each of the individual pathways, including adipokine signaling, gut microbiota regulation, or amino acid metabolism separately, there is no detailed description of how diet-sensitive metabolic pathways are connected to inflammatory signaling and the severity of the disease. Also, there is a lack of translational evidence linking advanced metabolomics and microbiome results to current clinical approaches to nutritional interventions. According to Araujo et al. (9), gut-pancreas interactions are not incorporated into predictive and therapeutic models, particularly in the acute inflammatory conditions of AP.

The main aim of the current research is to examine how nutritional modulation can be used to influence disease severity in patients with acute pancreatitis, considering clinical, metabolic, and inflammatory approaches. In particular, the research will assess the relationship between initial nutritional state and the severity of acute pancreatitis, and examine major diet-sensitive metabolic pathways, particularly amino acid and lipid metabolism, that contribute to disease development. Moreover, the study aims to clarify the role of inflammatory signaling pathways involving nutrition, the gut microbiota, and pancreatic injury. Lastly, the study aims to evaluate the influence of nutrition-related factors on clinically relevant outcomes such as feeding intolerance, complication rates, and recovery patterns, and, specifically, the long-term aim is to support a more mechanism-oriented and individualized nutritional strategy for the management of acute pancreatitis.

## 2 Methodology

### 2.1 Study design and population

This retrospective observational study of adult patients diagnosed with acute pancreatitis (AP) and admitted to the hospital between January 2021 and November 2025 was conducted. Inclusion criteria were age ≥18 years and confirmation of AP diagnosis according to the revised Atlanta classification. Patients with chronic pancreatitis, malignancy, pregnancy, or incomplete medical records were excluded. So, 1,600 patients were stratified by disease severity into mild, moderately severe, and severe AP. This study was approved by the Ethics Committee of the First Affiliated Hospital of Jinzhou Medical University, with approval number: KYLL2025320.

### 2.2 Data collection

Demographic, clinical, laboratory, and nutritional data were obtained from electronic medical records. The baseline variables were age, sex, BMI, comorbidities, etiology of AP, time to admission, and APACHE II score. The initial nutritional status was determined through serum biomarkers (albumin, prealbumin, vitamin D), anthropometric measures, and the NRS-2002 scoring system. In accordance with a careful dietary history, habitual food intake patterns were documented side by side. Data on patients’ routine medications for comorbid conditions (e.g., antidiabetic, lipid-lowering, or anti-inflammatory drugs) were not consistently available across all records due to the study’s retrospective design. Therefore, these variables were not included in the final analysis.

### 2.3 Nutritional intervention and feeding strategies

All available data on enteral nutrition (EN) were examined, taking into account not only the timing of EN initiation (early ≤48 h vs. delayed >48 h), but also the delivery route (nasogastric or nasojejunal), the type of formula used (standard, fiber-containing, immunonutrition), and the appointed caloric and protein targets along with the tolerance episodes.

### 2.4 Laboratory and biomarker assessment

Markers of inflammation (CRP, IL-6, TNF-*α*, IL-10, procalcitonin, neutrophil-to-lymphocyte ratio), metabolism (glucose, HOMA-IR, lactate, *β*-hydroxybutyrate, free fatty acids, TCA intermediates), and adipokines/immunometabolic mediators (leptin, adiponectin, resistin, FGF-21, MCP-1) were quantified during the first 72 h of patient admission to the hospital. Gut barrier integrity and microbiome biomarkers (zonulin, LPS, SCFAs, bacterial translocation) were also recorded whenever possible.

### 2.5 Clinical outcomes

The primary outcomes were disease severity progression, persistent organ failure, and in-hospital mortality. The secondary outcomes included the duration of hospital and ICU stay, complications, functional recovery, weight loss, and readmission rates.

### 2.6 Statistical analysis

Continuous variables were computed as mean ± standard deviation (SD) and then compared using ANOVA. Categorical variables were reported as counts and percentages and compared using the chi-square test. To derive independent nutritional factors for severe acute pancreatitis (AP), persistent organ failure, and death, a multivariable logistic regression model was applied, controlling for age, gender, BMI, reason, and other existing medical conditions. The odds ratios (ORs) with their corresponding 95% confidence intervals (CIs) were reported. A *p*-value of less than 0.05 was taken as statistically significant.

### 2.7 Ethical considerations

The research received approval from the Institutional Review Board/Ethics Committee, and the requirement for informed consent was waived due to the retrospective design of the study. All patient data were de-identified to ensure privacy.

## 3 Results

### 3.1 Baseline demographic and clinical characteristics of patients with acute pancreatitis

The patients in the severe AP group were older and had higher BMI to a significant extent in comparison with the mild and moderately severe groups. The ratio of males to females gradually increased with disease severity. The presence of alcohol-related etiology, diabetes mellitus, hypertension, and smoking was more noticeable in the moderately severe and severe AP; meanwhile, gallstone etiology was more frequent in the mild AP. The duration of hospital admission was longest in the severe case. APACHE II scores showed a clear stepwise increase from mild to severe AP, indicating greater physiological derangement with increasing disease severity (Table 1).

**Table 1:** Baseline demographic and clinical characteristics (*n* = 1,600).

Parameter	Total (*n* = 1,600)	Mild AP (*n* = 720)	Moderately severe AP (*n* = 520)	Severe AP (*n* = 360)	*p*-value
Age (years)	53.4 ± 14.1	49.8 ± 13.0	54.6 ± 14.2	59.8 ± 15.3	<0.001
Male sex, *n* (%)	992 (62.0)	424 (58.9)	328 (63.1)	240 (66.7)	0.02
Female sex, *n* (%)	608 (38.0)	296 (41.1)	192 (36.9)	120 (33.3)	0.02
BMI (kg/m^2^)	28.1 ± 5.2	26.7 ± 4.6	28.6 ± 5.1	30.4 ± 5.6	<0.001
Alcohol etiology	652 (40.8)	256 (35.6)	224 (43.1)	172 (47.8)	<0.001
Gallstone etiology	724 (45.3)	368 (51.1)	228 (43.8)	128 (35.6)	<0.001
Diabetes mellitus	394 (24.6)	128 (17.8)	146 (28.1)	120 (33.3)	<0.001
Hypertension	686 (42.9)	276 (38.3)	228 (43.8)	182 (50.6)	<0.001
Smoking	578 (36.1)	232 (32.2)	188 (36.2)	158 (43.9)	<0.001
Time to admission (h)	19.4 ± 9.8	16.2 ± 8.1	20.4 ± 9.6	24.6 ± 10.8	<0.001
APACHE II score	10.6 ± 5.2	6.8 ± 3.1	11.2 ± 3.9	16.8 ± 5.1	<0.001

### 3.2 Nutritional status at admission across acute pancreatitis severity classes

Serum albumin and prealbumin, as indicators of nutritional reserve, were significantly and progressively reduced from mild to severe disease. The occurrence of vitamin D deficiency, hypertriglyceridemia, sarcopenia, and biochemical abnormalities like hypocalcemia and hypophosphatemia escalated successively with increasing severity. Nutritional risk assessed by NRS-2002 scores ≥3 and clinically diagnosed malnutrition were significantly predominant in severe cases. Weight loss (>5%) was also more frequent in higher severity categories. Statistically significant differences were found in all parameters, indicating a strong correlation between the decline in nutritional status and the severity of the disease (Table 2).

**Table 2:** Nutritional status at admission by severity class.

Parameter	Mild (*n* = 720)	Moderately severe (*n* = 520)	Severe (*n* = 360)	*p*-value
Albumin (g/dL)	3.72 ± 0.54	3.32 ± 0.61	2.92 ± 0.69	<0.001
Prealbumin (mg/dL)	22.8 ± 6.0	17.6 ± 5.8	12.9 ± 4.9	<0.001
Vitamin D deficiency	284 (39.4%)	292 (56.2%)	260 (72.2%)	<0.001
Hypertriglyceridemia	198 (27.5%)	204 (39.2%)	196 (54.4%)	<0.001
Sarcopenia	138 (19.2%)	170 (32.7%)	194 (53.9%)	<0.001
NRS-2002 ≥ 3	216 (30.0%)	280 (53.8%)	272 (75.6%)	<0.001
Hypocalcemia	196 (27.2%)	242 (46.5%)	224 (62.2%)	<0.001
Hypophosphatemia	96 (13.3%)	128 (24.6%)	134 (37.2%)	<0.001
Malnutrition diagnosis	144 (20.0%)	202 (38.8%)	218 (60.6%)	<0.001
Weight loss >5%	122 (16.9%)	166 (31.9%)	198 (55.0%)	<0.001

### 3.3 Characteristics of nutritional interventions according to timing of enteral nutrition

The early EN intervention was significantly earlier and predominantly conducted through the nasogastric route, while the delayed EN intervention required nasojejunal feeding more often. The early EN patients were able to maintain oral intake more often and reached higher caloric objectives on day 3, where they had significantly more protein and fat consumed. The administration of fiber and immunonutrition was more common in the early EN group. Conversely, the late feeding to be poorly tolerated was noted substantially more frequently in the delayed EN group. All the distinctions were found to be statistically significant and pointed out the clinical advantages of early-start enteral nutrition (Table 3).

**Table 3:** Nutritional intervention characteristics.

Parameter	Early EN (*n* = 920)	Delayed EN (*n* = 680)	*p*-value
Time to feeding (h)	21.6 ± 6.8	63.9 ± 15.4	<0.001
Nasogastric feeding	592 (64.3%)	336 (49.4%)	<0.001
Nasojejunal feeding	328 (35.7%)	344 (50.6%)	<0.001
Oral tolerance achieved	716 (77.8%)	392 (57.6%)	<0.001
Caloric target day 3	81.4 ± 15.2%	54.8 ± 18.6%	<0.001
Protein intake (g/kg/d)	1.12 ± 0.29	0.78 ± 0.32	<0.001
Lipid calories (%)	26.8 ± 6.6	18.2 ± 7.4	<0.001
Fiber-containing formula	468 (50.9%)	182 (26.8%)	<0.001
Immunonutrition	306 (33.3%)	144 (21.2%)	<0.001
Feeding intolerance	182 (19.8%)	292 (42.9%)	<0.001

### 3.4 Inflammatory biomarker profiles according to feeding strategy

Pro-inflammatory markers such as C-reactive protein, Il-6, TNF-*α*, procalcitonin, ferritin, and ESR were found to be significantly lower during early EN. Neutrophil counts and the neutrophil-to-lymphocyte ratio were very low while lymphocyte count and the anti-inflammatory cytokine IL-10 were high in the early EN group. On the other hand, delayed EN was marked by severe inflammatory reactions and greater immune dysfunction. The differences in biomarkers were not only statistically significant but also clinically relevant, thus confirming that early enteral feeding is linked to the reduction of systemic inflammation in acute pancreatitis (Table 4).

**Table 4:** Inflammatory biomarkers by feeding strategy.

Marker	Early EN (*n* = 920)	Delayed EN (*n* = 680)	*p*-value
CRP (mg/L)	88 ± 46	142 ± 62	<0.001
IL-6 (pg/mL)	29.6 ± 13.2	48.9 ± 19.6	<0.001
TNF-α (pg/mL)	20.1 ± 8.4	33.4 ± 12.8	<0.001
IL-10 (pg/mL)	15.1 ± 6.2	9.4 ± 4.8	<0.001
Neutrophils (×10^9^/L)	9.8 ± 3.4	13.1 ± 4.3	<0.001
Lymphocytes (×10^9^/L)	1.45 ± 0.52	0.94 ± 0.41	<0.001
NLR	7.1 ± 3.6	14.2 ± 5.6	<0.001
Procalcitonin (ng/mL)	0.66 ± 0.48	1.56 ± 0.82	<0.001
Ferritin (ng/mL)	492 ± 186	738 ± 254	<0.001
ESR (mm/h)	44 ± 17	66 ± 22	<0.001

### 3.5 Metabolic pathway biomarkers across acute pancreatitis severity

The metabolic stress markers and those of anaerobic metabolism, such as serum lactate and fasting glucose, showed a progressive increase from mild to severe disease. Insulin resistance, as indicated by rising HOMA-IR values, and enhanced lipolysis and ketogenesis, as shown by the elevations in free fatty acids and *β*-hydroxybutyrate, were most pronounced in severe AP. Conversely, the tricarboxylic acid cycle intermediates such as citrate decreased with the growing severity of the disease, while succinate build-up was indicative of mitochondrial dysfunction. Blood urea nitrogen and serum ammonia, which are renal and nitrogen metabolism markers, showed a significant increase. Energy expenditure was marked by a progressive increase, which was suggestive of the rampant hypermetabolism that accompanied disease severity (Table 5).

**Table 5:** Metabolic pathway biomarkers by severity.

Parameter	Mild AP (*n* = 720)	Moderately severe AP (*n* = 520)	Severe AP (*n* = 360)	*p*-value
Serum lactate (mmol/L)	1.6 ± 0.4	2.3 ± 0.6	3.2 ± 0.9	<0.001
Fasting glucose (mg/dL)	116 ± 26	148 ± 38	186 ± 52	<0.001
HOMA-IR	2.0 ± 0.9	3.5 ± 1.3	5.3 ± 2.1	<0.001
β-hydroxybutyrate (mmol/L)	0.34 ± 0.15	0.59 ± 0.23	0.86 ± 0.36	<0.001
Free fatty acids (mmol/L)	0.47 ± 0.18	0.74 ± 0.26	1.04 ± 0.34	<0.001
Citrate (μmol/L)	126 ± 29	97 ± 27	71 ± 25	<0.001
Succinate (μmol/L)	43 ± 12	62 ± 15	89 ± 21	<0.001
Blood urea nitrogen (mg/dL)	17 ± 6	27 ± 10	41 ± 15	<0.001
Serum ammonia (μmol/L)	35 ± 10	49 ± 13	68 ± 19	<0.001
Energy expenditure (kcal/day)	1,960 ± 270	2,260 ± 330	2,610 ± 390	<0.001

### 3.6 Adipokine and immunometabolic mediator profiles across acute pancreatitis severity

According to Table 6, a clear pattern of changes in the profile of adipokines and immunometabolic mediators depending on the severity of the disease was observed among the patients diagnosed with acute pancreatitis. All the pro-inflammatory and stress-related markers, namely, leptin, resistin, MCP-1, PAI-1, and FGF-21, were seen to cumulatively rise as the disease progressed from mild to severe. On the other hand, the anti-inflammatory and metabolically favorable ones like adiponectin, omentin, irisin, and ghrelin were marked to severely decline as the severity of the disease increased. The CRP/adiponectin ratio showed a steep increase across the severity categories reflecting a transition to a pro-inflammatory immunometabolic state. These findings are both consistent and statistically significant indicating very tightly linked adipose tissue signaling, metabolic dysregulation, and inflammatory burden in the case of severe acute pancreatitis.

**Table 6:** Adipokines and immunometabolic mediators by severity.

Marker	Mild AP (*n* = 720)	Moderately severe AP (*n* = 520)	Severe AP (*n* = 360)	*p*-value
Leptin (ng/mL)	12.8 ± 6.4	19.6 ± 8.7	27.8 ± 10.8	<0.001
Adiponectin (μg/mL)	9.8 ± 3.2	7.1 ± 2.9	5.0 ± 2.5	<0.001
Omentin (ng/mL)	392 ± 96	318 ± 88	244 ± 76	<0.001
Resistin (ng/mL)	6.9 ± 2.5	9.8 ± 3.4	14.2 ± 4.9	<0.001
Ghrelin (pg/mL)	418 ± 112	346 ± 98	268 ± 91	<0.001
FGF-21 (pg/mL)	232 ± 78	352 ± 118	526 ± 172	<0.001
Irisin (ng/mL)	7.6 ± 2.2	5.8 ± 1.9	4.0 ± 1.7	<0.001
MCP-1 (pg/mL)	190 ± 68	284 ± 96	402 ± 132	<0.001
PAI-1 (ng/mL)	21.8 ± 6.6	32.4 ± 9.4	45.3 ± 13.6	<0.001
CRP/adiponectin ratio	8.4 ± 4.2	18.9 ± 8.4	34.1 ± 14.3	<0.001

### 3.7 Association of habitual dietary patterns with risk of severe acute pancreatitis

Table 7 presents habitual dietary exposures and associated risk estimates for non-severe versus severe acute pancreatitis (AP). High-fat, refined sugar, ultra-processed food, low-protein, and low-fiber intake diets were excessively common among severe AP cases and were linked to the doubling or tripling of the non-severe disease risk. Heavy drinking contributed to the severity of the disease too. On the other hand, omega-3-rich diets, Mediterranean diet, adequate vitamin D, and antioxidants were dietary patterns that provided protection against severe AP and were found to be much less common among the patients with severe AP showing thereby strong association with the greatly reduced odds of the severe disease. The indispensable role of the habitual diet in determining the severity of AP is underlined by these results.

**Table 7:** Habitual dietary patterns and risk of severe AP.

Dietary exposure	Non-severe AP (*n* = 1,240)	Severe AP (*n* = 360)	OR (95% CI)	*p*-value
High-fat diet	432 (34.8%)	198 (55.0%)	2.29 (1.79–2.93)	<0.001
Low protein intake	452 (36.5%)	228 (63.3%)	3.00 (2.35–3.83)	<0.001
Low fiber intake	496 (40.0%)	244 (67.8%)	3.14 (2.46–4.00)	<0.001
High refined sugar	568 (45.8%)	258 (71.7%)	2.98 (2.30–3.87)	<0.001
Omega-3 rich diet	518 (41.8%)	92 (25.6%)	0.48 (0.36–0.64)	<0.001
Mediterranean diet adherence	486 (39.2%)	78 (21.7%)	0.43 (0.32–0.59)	<0.001
Adequate vitamin D	612 (49.4%)	96 (26.7%)	0.37 (0.28–0.49)	<0.001
Antioxidant-rich diet	584 (47.1%)	104 (28.9%)	0.46 (0.35–0.61)	<0.001
Excess alcohol intake	412 (33.2%)	190 (52.8%)	2.25 (1.76–2.89)	<0.001
Ultra-processed foods	548 (44.2%)	262 (72.8%)	3.39 (2.60–4.42)	<0.001

### 3.8 Gut barrier dysfunction and microbial profiles according to feeding strategy

The comparison of gut barrier integrity and microbial markers in patients receiving early versus delayed enteral nutrition (EN) is presented in Table 8. It was found that early EN decreased the amount of zonulin, endotoxin activity, lipopolysaccharide concentrations, and fecal calprotectin significantly indicating the better preservation of intestinal barrier function. The proportions of beneficial microbial taxa, such as Bifidobacterium and Lactobacillus, were greater with early EN, which also resulted in the production of more short-chain fatty acids. On the other hand, delayed EN resulted in increased Enterobacteriaceae prevalence, higher rates of bacterial translocation, and infected pancreatic necrosis. All the differences were statistically significant, thus supporting the view that early enteral feeding is crucial for gut integrity and infectious complications control.

**Table 8:** Gut barrier dysfunction and microbial markers by feeding strategy.

Parameter	Early EN (*n* = 920)	Delayed EN (*n* = 680)	*p*-value
Zonulin (ng/mL)	44 ± 16	70 ± 24	<0.001
Lipopolysaccharide (EU/mL)	0.44 ± 0.19	0.82 ± 0.34	<0.001
Endotoxin activity (%)	29 ± 10	48 ± 15	<0.001
Bifidobacterium abundance	Higher	Lower	<0.001
Lactobacillus abundance	Higher	Lower	<0.001
Enterobacteriaceae dominance	Lower	Higher	<0.001
SCFA concentration (μmol/L)	84 ± 26	48 ± 21	<0.001
Fecal calprotectin (μg/g)	132 ± 68	296 ± 124	<0.001
Bacterial translocation (%)	7.4	21.3	<0.001
Infected necrosis (%)	9.1	24.7	<0.001

### 3.9 Analysis of nutritional predictors of persistent organ failure in acute pancreatitis

**Figure 1:**
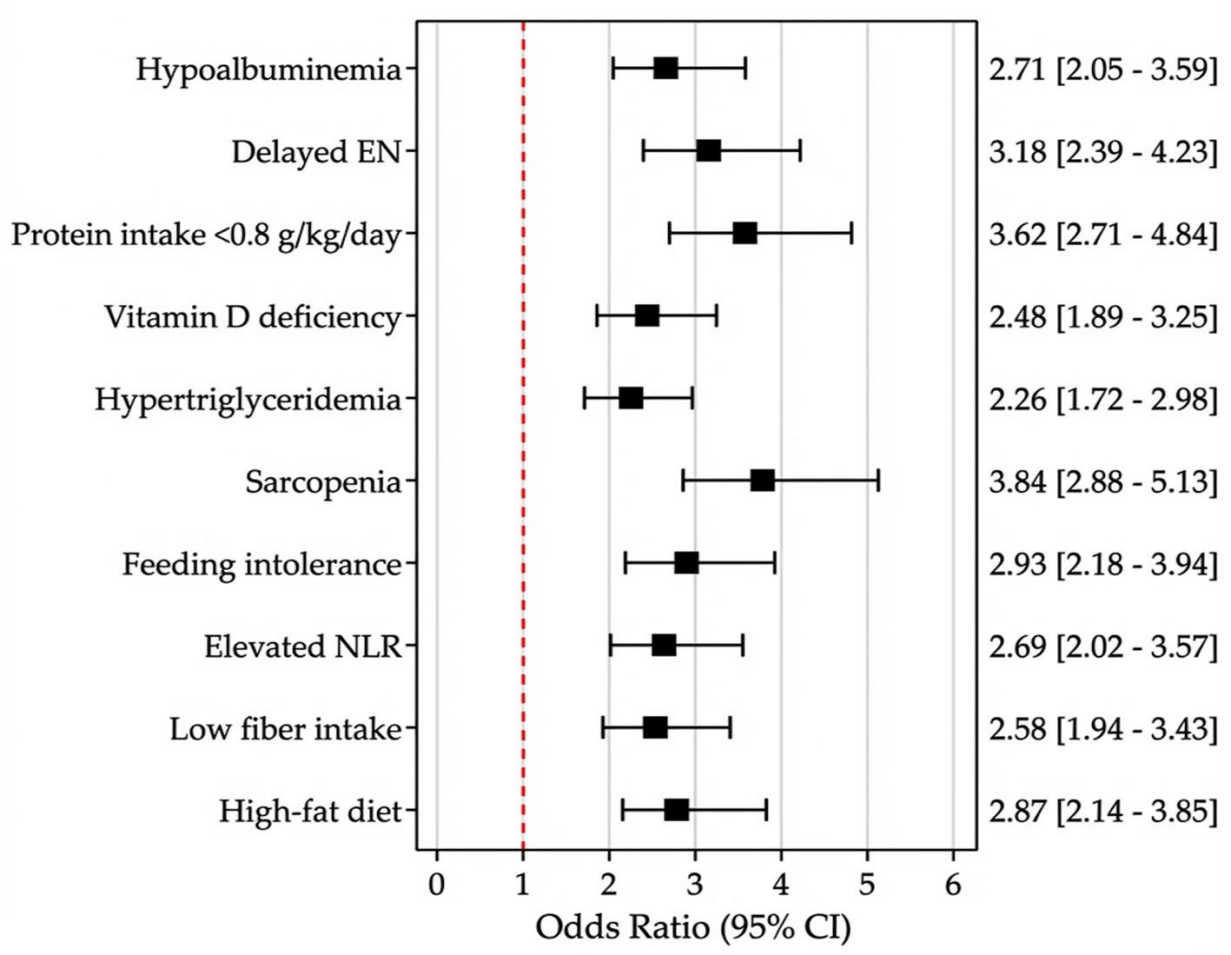
Nutritional predictors of persistent organ failure.

The analysis of nutritional predictors of persistent organ failure in acute pancreatitis revealed that hypoalbuminemia, delayed initiation of enteral nutrition, inadequate protein intake, vitamin D deficiency, hypertriglyceridemia, and sarcopenia were the independent nutritional predictors linked to persistent organ failure in acute pancreatitis. Spectacularly high risk ratios were calculated from 2.26 to 3.84 after adjusting for confounders on a long list of variables populating the questionnaire. Furthermore, feeding intolerance, elevated neutrophil-to-lymphocyte ratio, low dietary fiber intake, and high-fat diet also significantly increased the likelihood of organ failure. Adjustment for demographic and clinical covariates did not change the significance of the associations, thus underlining the vital role of nutritional status and dietary factors in the progression of the disease (Figure 1).

### 3.10 Hospital stay and recovery outcomes by feeding strategy

Patients receiving early versus delayed enteral nutrition (EN) are the subjects of Table 9, which displays the comparison of their hospital stay and recovery outcomes. Early EN brought about a reduction in the overall hospital stay and ICU stay periods by a great deal and a decrease in the rates of ICU admission, faster pain resolution, CRP normalization, and resumption of oral diet. The early EN group had local complications, readmissions, and weight loss that were very much lower than the other groups. Discharge functional recovery was significantly increased by early EN (83.6% vs. 60.7%). All the differences were statistically significant, implying that timely starting of enteral nutrition dramatically improves clinical recovery, lowers complications, and raises total functional outcomes in acute pancreatitis.

**Table 9:** Hospital stay and recovery outcomes.

Outcome	Early EN (*n* = 920)	Delayed EN (*n* = 680)	*p*-value
Total hospital stay (days)	9.8 ± 3.6	16.4 ± 6.5	<0.001
ICU admission (%)	174 (18.9%)	268 (39.4%)	<0.001
ICU stay duration (days)	4.3 ± 2.1	7.9 ± 3.5	<0.001
Pain resolution (days)	3.4 ± 1.3	5.9 ± 2.2	<0.001
CRP normalization (days)	5.3 ± 1.9	9.2 ± 3.1	<0.001
Oral diet resumption (days)	4.6 ± 1.8	8.6 ± 2.7	<0.001
Local complications (%)	212 (23.0%)	314 (46.2%)	<0.001
Readmission (%)	86 (9.3%)	144 (21.2%)	<0.001
Weight loss (%)	3.9 ± 2.2	7.6 ± 3.6	<0.001
Functional recovery (%)	83.6	60.7	<0.001

### 3.11 Multivariate analysis of nutritional modulators influencing disease severity in acute pancreatitis

The independent nutritional factors correlated with acute pancreatitis severity according to multivariable logistic regression are shown in Table 10. Early enteral nutrition, adequate protein intake, a diet rich in omega-3, and sufficient vitamin D were some of the protective factors that contributed to a considerable decrease in the likelihood of severe disease (adjusted ORs 0.44–0.53). On the contrary, high-fat diet, low fiber intake, sarcopenia, delayed feeding, and a combination of high neutrophil-to-lymphocyte ratio and hypoalbuminemia were among the factors that greatly (adjusted ORs 2.49–3.22) heightened the risk of developing severe disease. The associations were significant even after adjustments were made for age, sex, BMI, etiology, and comorbidities, thereby emphasizing the paramount role of nutritional status and dietary patterns in disease development.

**Table 10:** Multivariate regression: nutritional modulators of disease severity.

Variable	Adjusted OR	95% CI	*p*-value
Early EN	0.44	0.34–0.58	<0.001
Adequate protein intake	0.49	0.38–0.64	<0.001
Omega-3 rich diet	0.53	0.40–0.70	<0.001
Vitamin D sufficiency	0.47	0.36–0.62	<0.001
High-fat diet	2.78	2.11–3.67	<0.001
Low fiber intake	2.61	1.99–3.43	<0.001
Sarcopenia	3.22	2.41–4.30	<0.001
Delayed feeding	3.05	2.31–4.04	<0.001
Elevated NLR	2.68	2.02–3.56	<0.001
Hypoalbuminemia	2.49	1.89–3.29	<0.001

## 4 Discussion

The current research supplies good evidence that nutritional conditions, scheduling, and content of enteral nutrition, and related metabolic-inflammatory systems are closely associated with the severity of the disease and clinical results in acute pancreatitis (AP). These findings support and expand the existing literature by combining demographic, nutritional, biochemical, and inflammatory data from a large cohort and filling important mechanistic gaps. As it was done in earlier epidemiological research, patients with severe AP of the current cohort were also much older and had greater BMI than those with mild or moderately severe disease. The issue of obesity has been considered a factor in AP severity because it facilitates bone marrow lipotoxicity, inflammation of adipose tissue, and cytokine release. Ji et al. (13) showed using Mendelian randomization that genetic conditions associated with obesity cause increased inflammatory signaling and poor outcome in AP, which also supports our results that higher BMI is associated with severe disease phenotypes.

The steadily rising alcohol-related etiology, diabetes mellitus, hypertension and smoking in the categories of severity jointly emphasize the role of metabolic comorbidities in the development of diseases. On the other hand, the AP with the presence of gallstones was more common in mild cases, which is in line with previous findings that biliary AP tends to have an earlier onset and a more positive prognosis (1). The progressive increase in the APACHE II scores as the levels of systemic physiological derangement became more severe supports the validity of severity stratification in this group. One strength of this study is that the nutritional status at the time of admission was described in detail. The serum albumin and pre-albumin decreased gradually in mild and severe AP and indicated the reduced nutritional stores and increased inflammatory load. These results are quite consistent with the study by Genc et al., (14), who revealed that such nutritional checks as albumin-based scores are predictors of negative clinical outcomes in AP.

The growing rates of sarcopenia, weight loss, deficiency of vitamin D and hypertriglyceridemia in severe AP clearly indicate the multidimensional character of malnutrition among the population. There have been claims that hypertriglyceridemia and especially hypertriglyceridemia have been associated with worsening of pancreatic injury by inducing free fatty acid toxicity and mitochondrial dysfunction as evidenced by recent mechanistic studies (15, 16). The high rates of the patients having a score of NRS-2002 ≥ 3 and malnutrition clinically diagnosed among severe AP supports the idea that nutritional risk is not an effect but a cause of disease severity. Electrolyte imbalances including hypocalcemia and hypophosphatemia were also more common in more severe cases, and this was indicative of systemic inflammation, metabolic cellular changes, and excessive nutritional requirements. These biochemical abnormalities also highlight metabolic stress that is caused by the extreme pancreatic inflammation. The review of the nutritional interventions showed strong benefits of early enteral nutrition (EN). Patients that were given early EN fed much earlier, were able to tolerate oral intake more, and had reached higher caloric and protein goals by day three. These results are supported by the evidence of the foundational studies conducted by Olah and Romics (17) which showed that the early EN maintains gut integrity and infectious complications are less frequent than delayed feeding. Interestingly, a high level of nasogastric feeding, formulas that contain fiber and immune-nutrition were all linked with early EN. This is clinically pertinent, given that current evidence indicates that fiber and nutrients associated with enhancing immune functions alleviate the composition of gut microbiota and immune reactions. Yang et al. (18) demonstrated a beneficial effect of EN on the gut microbial diversity and decreasing inflammatory issues, which confirmed the preferable tolerance and metabolic sufficiency in our early EN group.

Conversely, delayed EN was associated with an increased rate of feeding intolerance, reduced nutrient delivery, and increased dependence on nasojejunal feeding. On its own, feeding intolerance has been found to be an indicator of gut dysfunction and extreme inflammation, which agrees with the previous findings that intolerance is a sign of impaired motility, dysbiosis, and hyper inflammatory cytokines. This study has been found to have one of the most interesting results through the significant difference between the inflammatory biomarker profile of early and delayed EN groups. There was a significantly reduced level of CRP, IL-6, TNF-*α*, procalcitonin, ferritin, and ESR, and better leukocyte profiles, such as reduced neutrophil counts and neutrophil to lymphocyte ratio (NLR), and increased lymphocyte counts in early EN. These results are very much in line with the mechanistic model suggested by Luo et al. (19), as NF-kB, cytokine cascades (2025), as NF-κB, cytokine cascades, and dysregulation of immune cells are core promoters of AP severity. The fa, and not IL-10 levels in the early EN group increased also indicates an anti-inflammatory response rather than merely the dampening of caloric deficits by nutritional intervention. Notably, McKenzie et al. (20) showed that enteral nutrition adjusts adipokine patterns in AP, causing a drop in pro-inflammatory mediators and enhancing metabolic signaling. Our results further extend this concept by indicating that early EN also positively alters classical inflammatory and immune biomarkers, thereby supporting the hypothesis that nutrition is a biological controller of inflammation rather than passive support.

The successive degradation of nutritional and inflammatory parameters observed in severe AP correlates with the new metabolomic findings. Amino acid metabolism was associated with immune activation and disease severity, and Yang et al. (21) defined dysregulated ornithine metabolism as one of the hallmarks of severe AP. These results are complementary to our findings on progressive protein-energy malnutrition and increased inflammation in severe cases. Also, in recent years, considerable attention has been paid to whether the gut microbiota and its metabolites influence inflammatory responses. Pan et al. and Guo et al. (16, 22) established that microbially produced short-chain fatty acids have anti-inflammatory protective effects, especially in hypertriglyceridemia-related AP. The elevated results for the fiber-containing formulas in the early EN group are possible because higher levels of these desirable metabolites were produced. The anti-inflammatory capacity of diet-derived bioactive compounds is further supported by Nani and Tehami (23), who emphasize the ability of polyphenols to suppress inflammasome activity. Although direct evaluation of polyphenols was not carried out in the study, the results collectively suggest a broader paradigm in which diet-sensitive metabolic and microbial pathways play a vital role in the severity of AP.

Gradually increasing disease severity was characterized by a progressive shift from oxidative to anaerobic metabolism, as evidenced by increases in serum lactate, fasting glucose, HOMA-IR, free fatty acids, and *β*-hydroxybutyrate. The results point to extreme insulin resistance, increased lipolysis, and pathological ketogenesis, which are the signs of metabolic reprogramming under the influence of stress. The simultaneous drop in citrate as succinate accumulated indicates an obstruction of the TCA cycle and mitochondrial dysfunction, which have been implicated in the amplification of inflammation via hypoxia-inducible and NF-κB-dependent signaling pathways. These findings are in line with Gao et al. (24), who pointed out that metabolic derangements in severe AP continue to perpetuate the inflammatory process by maintaining mitochondrial injury and immune cell stimulation. Significantly, elevated levels of blood urea and serum ammonia are indicative of catabolic nitrogen loss and renal-hepatic metabolic overload, which also fit the hypermetabolic syndrome of critical AP. The high expenditure of energy in the categories of severity supports the idea that severe AP is a hypercatabolic syndrome and requires early and sufficient nutritional intervention.

Mediators of adipose tissues revealed a remarkable imbalance with severity. Pro-inflammatory adipokines (leptin, resistin, MCP-1, PAI-1, and FGF-21) went up, whereas the anti-inflammatory, insulin-sensitizing ones (adiponectin, omentin, irisin, and ghrelin) went down. The drastic increase in the CRP/adiponectin ratio indicates a shift toward a markedly pro-inflammatory immunometabolic state. This trend aligns with that of Wang et al. (25), who revealed that the joint regulation of inflammatory cascades in AP by adipose tissue signaling and microbial metabolites occurs. The high level of FGF-21 is likely indicative of a compensatory process in response to metabolic stress, but its inability to respond to inflammation in severe AP highlights metabolic exhaustion. Insulin resistance and endothelial dysfunction are further increased by the loss of adiponectin and omentin, which increases systemic injury.

The close correlations between regular dietary exposures and AP severity indicate that the pre-morbid nutritional environment is a determinant that determines disease course. High fat, refined sugars and ultra-processed food diets and low protein and/or fiber intake were each independently linked to two- to three-fold odds of severe AP. These results confirm previous mechanistic studies that indicate that high-fat and ketogenic diets exacerbate lipotoxicity and exacerbate pancreatic inflammation (26). On the other hand, the diets rich in omega 3, Mediterranean type diets, sufficient status of vitamin D, and diets rich in antioxidants were very protective. The synthesis of the inflammatory mediators by Tang et al. (27) was reduced by n-3 fatty acids and enhanced the microcirculatory functioning in AP, which supports the mechanistic role of the protective relationships in the current study. Similarly, Coman et al. (28) underscored the effects of antioxidants in reducing the effects of oxidative stress, which probably plays a role in minimizing the pancreatic and systemic damage.

One of the main discoveries of this research is the significant influence of feeding strategy on intestinal integrity of the barriers and on the composition of intestinal microbes. Early EN was associated with significantly lower zonulin, endotoxin activity, lipopolysaccharide levels, and fecal calprotectin, indicating that intestinal permeability was maintained. At the same time, positive taxa like Bifidobacterium and Lactobacillus were boosted, and elevated levels of short-chain fatty acids (SCFA) were obtained. These findings are closely consistent with Song et al. and Wang et al. (25, 29), who demonstrated that gut microbiota function and metabolite production play central roles in recovery and immune regulation in AP. The increase in SCFA levels, especially butyrate, promotes mucosal integrity and anti-inflammatory signaling, which is in line with the results of Li et al. (30), who demonstrated that dietary inulin alleviates severe AP through the gut-pancreas axis. Delayed EN, on the contrary, was linked to Enterobacteriaceae preponderance, augmented bacterial translocation, and augmented disease of pancreatic necrosis, which highlights the pathogenic effect of gut dysbiosis in the absence of enteral stimulation.

Hypoalbuminemia, delayed EN, inadequate protein intake, vitamin D deficiency, hypertriglyceridemia, sarcopenia, feeding intolerance, elevated NLR, low fiber intake, and high-fat diets were identified as independent predictors of persistent organ failure in multivariable analysis. These results underline that inflammatory load as well as nutritional stores are at the center stage of organ dysfunction in AP. The size of the risk factors for sarcopenia and delayed feeding is especially interesting, and it confirms that muscle mass and early nutritional intake are vital determinants of metabolic resilience. The previous studies by Ramanathan and Aadam and Lakananurak and Gramlich (31, 32) supported the idea of early EN on a clinical basis; the current study goes further and substantiates the concept at the biological and mechanistic levels. Early EN was associated with shorter hospital and ICU stays, lower readmission rates, fewer ICU admissions, faster pain management, earlier CRP restoration, fewer complications, lower readmission rates, and better functional recovery. Such results are similar to Liu et al. (33), who showed a favorable prognosis in severe AP with prebiotic-enriched EN. They once again confirm the idea that nutrition therapy plays an active role in altering the pathophysiology of the disease that has been put forward by Gao et al. (25).

### 4.1 Strengths of the study

The present research possesses a number of strengths that enhance the robustness and clinical applicability of its results. First, the sample size of 1,600 patients is quite large and provides strong statistical power to stratify across all severity categories of acute pancreatitis and to perform comprehensive multivariate analyses. Second, the revised Atlanta classification results in the use of standard and internationally accepted evaluation of disease severity, making it easier to compare with existing literature. Third, the research uses an integrative design by assessing nutritional status, metabolic reprogramming, inflammatory signaling, adipokine profiles, gut barrier health, and habitual eating habits in a single cohort, a design that is rarely used in a single cohort. This multidimensional test can be interpreted mechanistically rather than descriptively.

The other significant strength is the detailed description of enteral nutrition practices, including timing, route, composition, caloric and protein adequacy, and feeding tolerance. Such a degree of granularity makes it possible to conduct meaningful comparisons between early and delayed feeding strategies and provides real-world evidence to inform clinical decision-making. Moreover, the addition of gut barrier and microbiome-related markers can provide new insights into the gut-pancreas axis, enhancing the biological plausibility of the identified relationships. The independence and validity of the identified nutritional predictors of disease severity and organ failure are further supported by the adjustment of critical demographic and clinical confounders in the multivariate regression models.

### 4.2 Limitations of the study

Alongside its strengths, there are various shortcomings that should be recognized. The nature of the retrospective observational design is that the study cannot be causal, and the associations between nutritional factors and disease outcomes cannot be conclusively directional. Second, the use of electronic medical records poses a risk of biased information, especially in dietary history, feeding tolerance reports, and anthropometric measurements. Third, despite the close attention to documenting dietary habits, these were based on patient recollection or proxy reports, which are prone to recall bias and misclassification. Also, the results of biomarker measurements were mostly limited to the first 72 h of hospitalization, which does not allow tracking the dynamic longitudinal activity of metabolic and inflammatory biomarkers throughout the course of the disease. Not all patients were able to be tested with the microbiome and gut barrier tests, which could create selection bias and restrict the generalizability of those results. The other research limitation is that, due to differences in institutional feeding guidelines, clinicians’ preferences, and the supply of formulas during the study period, the nutritional practices may have been affected by these changes. The study is limited to a single cente; thus, its external validity is limited to non-clinical contexts where nutritional management or patient populations differ. An important limitation of this study is the lack of detailed information regarding concomitant medications used by patients for other comorbid conditions. Such medications, including antidiabetic agents, statins, and anti-inflammatory drugs, may influence metabolic and inflammatory parameters and potentially affect nutritional status and disease severity. Due to incomplete and inconsistent documentation in retrospective records, these factors could not be systematically analyzed.

### 4.3 Future directions

Prospective, multicenter cohort studies and randomized controlled trials should also be prioritized in future research to establish the causal role of nutritional modulation in altering disease severity and outcomes in acute pancreatitis. It would be of greater interest to profile metabolic, inflammatory, and adipokine markers over an extended period to gain a better understanding of the temporal relationships and mechanisms. Novel therapeutic targets could be identified, and advanced multi-omics methodologies (metabolomics, lipidomics, and microbiome sequencing) can help further elucidate nutrition-induced immunometabolic responses. Moreover, future research should also consider individualized nutrition plans based on metabolic patterns, body structure, and inflammatory states, such as accurate protein dosing, omega-3 fatty acid supplementation, prebiotics in the form of fiber, and optimizing vitamin D levels. Gut-directed nutritional trials, such as synbiotics and immunonutrition, should be encouraged to reduce the incidence of infectious complications and organ failure. Lastly, preventive scoring systems should incorporate nutritional risk assessment to enhance early risk stratification and predictive nutritional intervention in acute pancreatitis.

## 5 Conclusion

Nutritional modulation is one of the most significant factors affecting disease severity, inflammatory response, and clinical outcomes in acute pancreatitis. Enteral nutrition started very early, adequate protein intake, and diet patterns rich in omega-3 fatty acids, fiber, and antioxidants have strong associations with a reduction in systemic inflammation, maintaining gut barrier integrity, and also with a lower incidence of organ failure, shorter hospital stays, and better functional recovery. On the other hand, delayed feeding, high-fat or low-fiber diets, sarcopenia, hypoalbuminemia, and vitamin D deficiency are major factors that lead to the severe disease, persistent organ failure, and in-hospital mortality. The incorporation of metabolically and immunologically optimized nutritional strategies into standard AP management could significantly reduce disease progression, improve recovery trajectories, and enhance overall patient outcomes. Future research should focus on prospective designs incorporating detailed clinical data, including medication history and dietary patterns, to better understand their combined impact on disease severity and clinical outcomes.

## Data Availability

The raw data supporting the conclusions of this article will be made available by the authors, without undue reservation.
